# Gestational diabetes mellitus (GDM) and adverse pregnancy outcome in South Asia: A systematic review

**DOI:** 10.1002/edm2.285

**Published:** 2021-07-03

**Authors:** Sabuj Kanti Mistry, Rajat Das Gupta, Sabiha Alam, Kuljeet Kaur, Abu Ahmed Shamim, Shuby Puthussery

**Affiliations:** ^1^ BRAC James P Grant School of Public Health BRAC University Dhaka Bangladesh; ^2^ Centre for Primary Health Care and Equity UNSW Sydney NSW Australia; ^3^ Institute of Nutrition and Food Science University of Dhaka Dhaka Bangladesh; ^4^ Independent researcher Melbourne Vic. Australia; ^5^ Maternal and Child Health Research Centre Institute for Health Research University of Bedfordshire Luton UK

**Keywords:** adverse pregnancy outcomes, GDM, gestational diabetes mellitus, South Asia, systematic review

## Abstract

**Introduction:**

The prevalence of gestational diabetes mellitus (GDM) is increasing in developing countries including the South Asian Nations. The current study aimed to examine the association of GDM with adverse pregnancy outcomes from foetal and maternal perspectives in South Asia.

**Methods:**

A systematic review was conducted including primary studies published since January 2020 from South Asian countries. Following electronic databases were searched to locate the articles: MEDLINE, EMBASE and EMCARE. Data were extracted using a customized extraction tool and methodological quality of the included studies was assessed using modified Effective Public Health Practice Project (EPHPP) quality assessment tool. Narrative synthesis was performed as statistical pooling was not possible due to the heterogeneous nature of the studies.

**Results:**

Eight studies were included in the review. Overall, the review found a positive correlation between GDM and adverse foetal outcomes such as macrosomia, neonatal hyperglycaemia, intrauterine growth retardation (IUGR), stillbirths and low birthweight (LBW), but the findings were not conclusive. GDM was also positively associated with preeclampsia but the association between GDM and C‐section delivery was not conclusive.

**Conclusion:**

Policymakers, public health practitioners and researchers in South Asia should take in to account the link between GDM and adverse pregnancy outcomes while designing interventions to promote maternal health in South Asia. Researchers should focus on conducting longitudinal studies in future to clearly understand the epidemiology and pathobiology of this issue.

## Graphical abstract

This systematic review was conducted to examine the association of gestational diabetes mellitus (GDM) with adverse foetal and maternal outcomes in South Asian countries. A total of eight studies were included in the final review Overall, the review found a positive association between GDM and adverse foetal outcomes such as macrosomia, neonatal hyperglycaemia, intrauterine growth retardation (IUGR), stillbirths and low birth weight (LBW). The GDM was also found to be associated with adverse maternal outcomes such as preeclampsia and C‐section delivery.

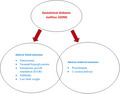

## 1 INTRODUCTION

Gestational diabetes mellitus (GDM) can be defined as glucose intolerance of varying degree started at or detected during pregnancy. GDM is known as one of the leading causes of maternal and infant mortality.^1^, ^2^ Studies have detected several risk factors of GDM including overweight, obesity, advanced maternal age and family history of diabetes.^3^, ^4^, ^5^

Globally, the prevalence of GDM is increasing in recent years and affects 1%–14% of all pregnancies.^6^ GDM was previously considered to be a major public health problem in developed countries, but it is now a growing problem in developing countries as well. Most of the countries in the South Asian region are no exception to this with an increasing trend in the prevalence of GDM reported in these countries.^7^ Evidence suggests that the prevalence of GDM is 11% higher among women from Indian subcontinent than those from Europe.^8^ Among Asian countries, the highest prevalence rate is reported in China and India.^9^ It is postulated that increased population density followed by the emergence of agriculture, regular famines and the retarded growth with thin physique characterization has elevated the general susceptibility to diabetes in Indian sub‐continent.^10^

Along with the increasing probability of being diagnosed with Type 2 diabetes mellitus after pregnancy, GDM is indicated to be closely associated with different adverse pregnancy outcomes both at the foetal and maternal level including an increased risk of caesarean‐section (C‐section) delivery, preeclampsia, macrosomia and intrauterine growth retardation (IUGR).^11^, ^12^, ^13^ GDM is also associated with newborns delayed brain maturity and neurobehavioral abnormalities including comparatively lower intelligence than normal babies, language impairments, poor attention and impulsivity.^14^ Therefore, it is very important that GDM is managed properly to avoid different adverse pregnancy outcomes associated with it.

Evidence suggests that the prevalence of adverse pregnancy outcomes such as preeclampsia, C‐section delivery, macrosomia, low birthweight, still births and IUGR is high in South Asian countries.^15^, ^16^, ^17^, ^18^ For example, Poudel et al. (2020)^17^ reported that the pooled stillbirth rate in India, Bangladesh, Nepal and Pakistan was 25.15 per 1,000 births. Another study reported that, the prevalence of C‐section delivery is around 13% in South Asian countries.^19^ Although several studies have pointed the association between GDM and adverse pregnancy outcomes, it is important that these findings are synthesized systematically to provide concrete evidence for policymakers and practitioners in South Asia. This study aimed to synthesize the evidence on the association between GDM and adverse pregnancy outcomes in South Asian countries.

## 2 METHODS

### 2.1 Data sources

We followed PRISMA (preferred reporting items for systematic reviews and meta‐analyses) guidelines for conducting this review.^20^ A systematic search of electronic databases such as MEDLINE, EMBASE and EMCARE was performed to identify the primary studies published since January 2020 from India, Bangladesh, Pakistan, Nepal, Bhutan, Sri Lanka, Maldives and Afghanistan. A combination of both MeSH terms and keywords was used to search the relevant articles such as: ‘Gestational diabetes mellitus’, ‘GDM’, ‘Neonatal hyperglycemia’, ‘Macrosomia’, ‘Stillbirth’, ‘Preeclampsia’, ‘Caesarean Section’, ‘Intrauterine growth retardation’, ‘Low birth weight’, ‘India’, ‘Bangladesh’, ‘Pakistan’, ‘Nepal’, ‘Bhutan’, ‘Sri Lanka’, ‘Maldives’ and ‘Afghanistan’. A Boolean searching technique was employed where ‘AND’, ‘OR’ and ‘NOT’ were used to unite different search terms.

### 2.2 Study selection

Selection of the articles was based on the predefined inclusion/exclusion criteria (Table 1). The search and screening process of the articles were conducted independently by two researchers (SKM and KK). The consistency was ensured by a third researcher (RDG). The full‐text screening and selection were done by all three researchers. Any discrepancy was solved by consensus among the group.

**TABLE 1:** Inclusion and exclusion criteria for study selection

Inclusion criteria
1) Studies investigated the role of gestational diabetes mellitus and adverse pregnancy outcomes
2) Primary studies
3) Conducted in South Asian countries, that is, India, Bangladesh, Pakistan, Nepal, Bhutan, Sri Lanka, Maldives and Afghanistan
4) Published in English literature
5) Peer‐reviewed articles
6) Published since January 2020
Exclusion criteria
1) Studies did not investigate the role of gestational diabetes mellitus and adverse pregnancy outcomes
2) Not primary studies
3) Conducted in places other than South Asian countries, that is, India, Bangladesh, Pakistan, Nepal, Bhutan, Sri Lanka, Maldives and Afghanistan
4) Published in language other than English
5) Not peer‐reviewed articles

### 2.3 Data extraction and quality assessment

Data from each of the selected studies were extracted using a data extraction template. Information such as title, authors, publication year, country, study design, study population and key findings were extracted from each article and presented in the extraction form in Microsoft excel 2013.

Methodological quality of the selected articles was assessed using modified Effective Public Health Practice Project (EPHPP) quality assessment tool.^21^ Selected studies were evaluated against four methodological domains: study design and appropriateness of the outcome measure, sample representativeness, data analysis and interpretation. In each individual study, for every domain, a score was assigned: 1 (weak), 2 (moderate) or 3 (strong). The score was then combined to get the total methodological quality score assigned to an individual article. A score of 4–6, 7–9 and 10–12 were graded as weak, moderate and strong respectively.^21^

### 2.4 Data synthesis

Data synthesis involved systematic collating, combining and summarizing the findings of the selected literatures to answer the research question of the review.^22^ Data analysis can be accomplished either using statistical pooling or where it is inappropriate by best evidence synthesis approach. Considering the heterogeneous nature of the included studies, the findings of the included studies were analysed as best evidence synthesis instead of statistical pooling.

## 3 RESULTS

### 3.1 Literature selection

**FIGURE 1:**
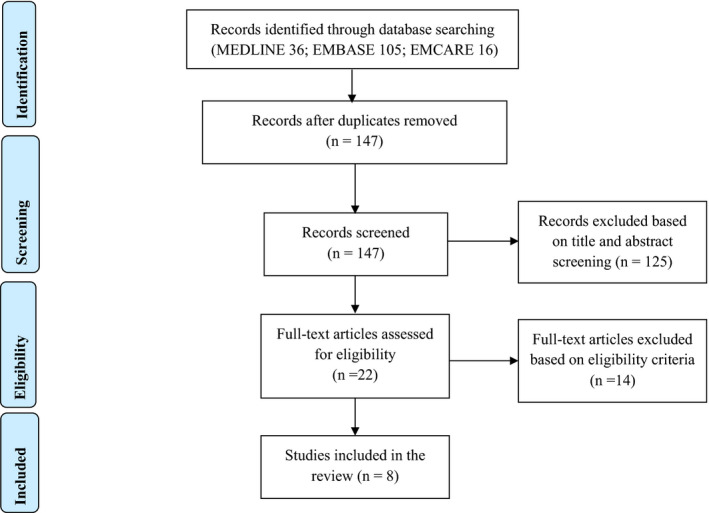
PRISMA flow diagram of the study selection process for inclusion in the review

A total of 157 articles were identified through initial searching of the electronic databases, of which 10 articles were excluded as duplicate records (Table 1). From the remaining 147 articles, screening of the title and abstracts based on the inclusion criteria resulted in exclusion of 125 articles. A total of 22 articles underwent full‐text screening, of which 14 articles were excluded based on the following reasons: not investigating the role of GDM on adverse pregnancy outcome and not a primary study. Eight articles were included in the final analysis (Figure 1).

### 3.2 Characteristics of included studies

Characteristics of included studies are shown in Table 2. Among the eight studies included,^8^, ^9^, ^23^, ^24^, ^25^, ^26^, ^27^, ^28^ six studies^8^, ^24^, ^25^, ^26^, ^27^, ^28^ utilized prospective observational study design while the remaining two studies^9^, ^23^ used a retrospective study design. All the studies selected were primary studies carried out in South Asian countries. Five studies were conducted in India,^8^, ^9^, ^26^, ^27^, ^28^ one in Pakistan,^23^ one in Bangladesh^24^ and one in Sri Lanka.^25^ All the studies were conducted in hospital settings except one study which was carried out in rural areas of Assam, India.^26^ In five studies, GDM was diagnosed in accordance with the WHO criteria and was measured performing an oral glucose tolerance test (OGTT),^9^, ^23^, ^25^, ^26^, ^27^ while in other studies (*n* = 3), patient recall were used.^8^, ^24^, ^28^

**TABLE 2:** Characteristics of included studies

Study	Title	Country	Study setting	Study design	Study population/subject/participants	Methodological quality
Akhter et al. (1996)^23^	Diabetes in Pregnancy in Pakistani Women: Prevalence and Complications in an Indigenous South Asian Community	Pakistan	Aga Khan University Hospital in Karachi, Pakistan	Retrospective study	6,830 pregnant women	Moderate
Islam et al. (2016)^24^	Morbidities and Mortalities Among Infant of Diabetic Mother in a Newly Established SCANU of a Tertiary Care Hospital, Bangladesh	Bangladesh	Mymensingh Medical College Hospital (MMCH), Bangladesh	Prospective observational study	A total 50 infant of diabetic mother (IDM) patients who were admitted during Jan and Mar 2015 were recruited in the study.	Weak
Jayawardane et al. (2016)^25^	Hyperglycemia in pregnancy among South Asian women: A single tertiary care center experience from Colombo, Sri Lanka	Sri Lanka	A tertiary care referral centre, Colombo, Sri Lanka	Prospective observational study	Data from first trimester to delivery of 572 were analysed	Moderate
Mahanta et al. (2014)^26^	Maternal and foetal outcome of gestational diabetes mellitus in a rural block of Assam, India	India	Rural areas in Dibrugarh District of Assam, India	Prospective observational study	A total of 930 antenatal women coming during Jun to August 2011 to subcentres, outreach areas, tea‐garden hospitals, state dispensaries, primary health centres and community health centres were included in the study.	Weak
Misra and Das (2017)^8^	Neonatal Outcome in Early and Late Gestational Diabetes Mellitus	India	A neonatal intensive care unit of a tertiary hospital in Kolkata, India	Prospective observational study	A total of 2,554 mothers delivered in the hospital were included in the study	Moderate
Saxena et al. (2011)^9^	Pregnancy Outcome of Women with Gestational Diabetes in a Tertiary Level Hospital of North India	India	A tertiary‐level hospital of North India	A retrospective analytical record‐based study	100 pregnant women (50 diabetic and 50 normoglycaemic)	Moderate
Shefali et al. (2006)^27^	Pregnancy Outcomes in Pre‐gestational and Gestational Diabetic Women in Comparison to Non‐diabetic Women–A Prospective Study in Asian Indian Mothers (CURES−35)	India	Dr. Mohan's Diabetes Specialities Centre, a tertiary care centre for diabetes in Chennai in southern India	Prospective observational study	225 patients were included from the health centre while 30 non‐diabetic controls were recruited from the ongoing population‐based study, the Chennai Urban Rural Epidemiology Study (CURES).	Moderate
Wahi et al. (2011)^28^	Prevalence of Gestational Diabetes Mellitus (GDM) and its Outcomes in Jammu Region	India	Department of Gynaecology and Obstetrics, SMGS Hospital and Department of General Medicine, Government Medical College and Hospital, Jammu	Prospective observational study	A total of 272 subjects at 24th to 28th weeks of gestation were evaluated for the GDM and outcome of GDM	Moderate

### 3.3 Methodological qualities of included studies

Methodological quality of the included studies was ranked against the predefined criteria and six^8^, ^9^, ^23^, ^25^, ^27^, ^28^ of the eight studies were classified as of moderate quality while two studies^24^, ^26^ were of weak methodological quality.

### 3.4 Narrative synthesis

Narrative synthesis of the selected articles was performed since the findings are very heterogeneous in nature. Overall, selected studies revealed that GDM has a role in adverse pregnancy outcomes both among the foetus such as neonatal hyperglycaemia, macrosomia, stillbirths, intrauterine growth retardation (IUGR) and low birthweight (LBW) and mothers such as preeclampsia and C‐section. The findings are shown in Table 3.

**TABLE 3:** Association between gestational diabetes mellitus and adverse pregnancy outcomes

Study	Gestational diabetes mellitus						
	Neonatal hyperglycaemia	Macrosomia	Stillbirth	IUGR	LBW	Preeclampsia	Caesarean section
Akhter et al. (1996)^23^	NA	13% of the foetus was suffering from macrosomia	NA	IUGR was 7.1% of GDM affected women	NA	Prevalence of preeclampsia was 19.1% among the GDM patients	C‐section rate was 25.9% among the GDM patients
Islam et al. (2016)^24^	Neonatal hyperglycaemia was found among 40% infants of infant with diabetic mother (IDM)	Macrosomia was presented among 24% of the mother with IDM	NA	NA	NA	NA	NA
Jayawardane et al. (2016)^25^	NA	Relatively lower among GDM compared to Hyperglycaemia in early pregnancy (HIEP) group (18.7% vs. 33.7%, *p *< .01)	NA	NA	No significant difference observed in LBW prevalence among groups (*p *= .76)	Prevalence of hypertension in pregnancy was higher among GDM group compared to those with Hyperglycaemia in early pregnancy	No significant difference was observed among groups in terms of prevalence of C‐section
Mahanta et al. (2014)^26^	NA	Macrosomia was higher among GDM group compared to the non‐GDM group (32% vs. 0.7%)	NA	NA	NA	Pregnancy‐induced hypertension was higher among GDM group (OR: 2.82)	NA
Misra and Das (2017)^8^	NA	Macrosomia was not significant (only 4% in the GDM group)	NA	NA	LBW was prevalent in the GDM group (21.8%)	Prevalence of pregnancy‐induced hypertension was 20.8% among GDM group	NA
Saxena et al. (2011)^9^	NA	The prevalence of macrosomia was significantly higher in GDM group (28% vs. 0%, *p *= .001)	NA	IUGR was significantly different between two groups	NA	Pregnancy‐induced hypertension was significantly higher among GDM group (40% vs. 10%, *p *= .001)	C‐section delivery was significantly higher in GDM group (42% vs. 0%, *p *= .001)
Shefali et al. (2006)^27^	NA	Macrosomia was highly prevalent among the GDM patients compared to the non‐diabetic group (27.6% vs. 7.1%, *p *= .04)	NA	NA	Prevalence of LBW was significantly lower among the GDM group compared to the non‐diabetic group (8.2% vs. 14.3%)	NA	NA
Wahi et al. (2011)^28^	NA	Macrosomia was highly prevalent among the GDM patients compared to the non‐diabetic group (16.2% vs. 5.7%, *p *= .02)	Stillbirth was significantly higher among GDM group (4.8% vs. 0%, *p *= .02)	NA	LBW was relatively higher in control group but not statistically significant (14.2% vs. 8.2%)	Gestational hypertension was higher among the GDM group (6.4% vs. 0%, *p *< .01)	NA

#### 3.4.1 GDM and neonatal hyperglycaemia

Islam et al.^24^ presented the role of GDM on neonatal hyperglycaemia. The authors reported that 40% of the GDM mothers have an infant with hyperglycaemia in neonatal period. However, the study did not report the values in any control group making it difficult to draw any concrete conclusions.

#### 3.4.2 GDM and macrosomia

All the studies^8^, ^9^, ^23^, ^24^, ^25^, ^26^, ^27^, ^28^ investigated the role of GDM on macrosomia. Six studies^8^, ^9^, ^25^, ^26^, ^27^, ^28^ showed that higher percentage of pregnant women with GDM have a foetal outcome of macrosomia compared to women with no GDM. Akhter et al.^23^ and Islam et al.^24^ reported that macrosomia was highly prevalent among GDM mother, although the findings were inconclusive due to lack of a control group.

#### 3.4.3 GDM and LBW

Four^8^, ^25^, ^27^, ^28^ of the eight studies reported the association between GDM and LBW. However, discrepant findings were reported. Although Misra and Das^8^ reported the prevalence of LBW among the GDM group, the study did not report the association in relation to that of any control group. On the contrary, Jayawardane et al.^25^ and Wahi et al.^28^ reported that there was no significant association between GDM and LBW. Shefali et al.^27^ found that prevalence of LBW was higher among the control group compared to the GDM group.

#### 3.4.4 GDM and intrauterine growth retardation (IUGR)

Role of GDM on IUGR was measured by two studies^9^, ^23^ but the findings were not conclusive. Akhter et al.^23^ found that 7.1% of the GDM women have IUGR but did not report anything regarding the control group; thus, making it difficult to draw any conclusion. Saxena et al.^9^ also did not find any conclusive evidence on the association between GDM and IUGR.

#### 3.4.5 GDM and stillbirths

Only Wahi et al.^28^ investigated the association between GDM and adverse pregnancy outcomes in the form of stillbirths. The study reported that prevalence of GDM was significantly higher among the GDM group (*p *= .02) compared to the control group.

#### 3.4.6 GDM and preeclampsia

Six of the eight studies investigated the association between GDM and preeclampsia or pregnancy‐induced hypertension. Irrespective of the study setting, all the studies reported a positive association between GDM and preeclampsia. Although Akhter et al.^23^ and Misra and Das^8^ did not report the result in comparison to a control group, they reported that prevalence of preeclampsia was very high (20.8%) among the pregnant women with GDM. On the other hand, Jayawardane et al.,^25^ Mahanta et al.,^26^ Saxena et al.^9^ and Wahi et al.^28^ reported the association between GDM and preeclampsia in comparison to a control group and reported that GDM group had significantly higher occurrence of preeclampsia compared to that of the control group.

#### 3.4.7 GDM and C‐section delivery

Three studies^9^, ^23^, ^25^ investigated the role of GDM on C‐section delivery. While Saxena et al.^9^ reported a positive association between GDM and C‐section delivery, the other two studies^23^, ^25^ did not find any significant association.

## 4 DISCUSSION

This systematic review was aimed at finding the role of GDM on adverse pregnancy outcomes at both foetal and maternal level. Most of the studies included in the review followed a prospective observational design and were carried out in hospital settings in India. Overall, the study findings revealed that GDM was associated with foetal macrosomia. While some studies identified the association of GDM on other adverse foetal outcomes such as neonatal hyperglycaemia, LBW, stillbirths and IUGR, the evidence was not conclusive. Although the association between GDM and preeclampsia or pregnancy‐induced hypertension among pregnant mothers was identified, it is difficult to conclude the associations between GDM and C‐section delivery.

Consistent with the findings of the present review, other studies have affirmed that untreated GDM is often linked to foetal macrosomia (children with birthweight greater than 4,000 grams).^29^, ^30^ The major reason behind the increased risk of macrosomia among pregnant mother with GDM is the enhanced insulin resistance of the mother^31^ due to a higher amount of glucose passing through the placenta into foetal circulation. Consequently, this extra amount of glucose is stored as body fat in the foetus and causes macrosomia.^32^

Similar to the findings from our review, other studies have reported positive correlation among GDM, stillbirths and LBW.^33^, ^34^ The leading causes of stillbirths related to GDM are reported to be abnormalities of placenta, congenital malformations and IUGR.^35^ Foetal anaerobic metabolism with hypoxia and acidosis resulting from hyperglycaemia is a common metabolic cause of diabetes‐related stillbirth.^36^

Women with GDM can give birth to neonates with different metabolic disorders. Neonatal hypoglycaemia is one of them which results from hyperinsulinism leading to neurodevelopmental outcomes.^37^ Therefore, if GDM can be controlled, the prevalence rate of neonatal hypoglycaemia can also be controlled as they are interrelated.

Other studies have confirmed the correlation between GDM and adverse outcomes of the pregnant mothers such as preeclampsia and C‐section delivery.^38^, ^39^, ^40^ Preeclampsia is a common pregnancy‐related complication induced by GDM, characterized by high blood pressure resulted from increased insulin resistance.^41^ Hypertensive disorders can be increased two‐ to threefold in pregnancies due to high blood glucose level.^42^ However, several other maternal factors are also associated with preeclampsia including maternal cardiovascular disease, renal disease, overweight and obesity.^43^ A strong positive association was found between GDM and C‐section delivery in a study from China among pregnant women in Chengdu, Sichuan province.^44^ However, in our systematic review, although one study reported positive association, two studies did not find any positive association between C‐section delivery and GDM. More studies are required to have a conclusive evidence on this.

As the review highlights the importance of GDM in increasing the prevalence of adverse pregnancy outcomes, it is important that policymakers and practitioners emphasize more on the preventive measures of GDM not only for the sake of GDM alone but also to prevent adverse pregnancy outcomes simultaneously. There are not many direct causes of adverse pregnancy outcomes that are documented; therefore, controlling GDM can indirectly act as a preventive measure for adverse pregnancy outcomes as well. It is important that national and regional policies regarding the prevention and management of GDM also incorporate policies that embed GDM management for adverse pregnancy outcomes. Regional collaborations between different stakeholders need to be strengthened with shared understanding, planning and implementation to address the problem. For example, using the platform of the South Asian Association for Regional Cooperation (SAARC) in active engagement among policymakers and practitioners from these countries can be of value in this regard. The findings of the present review are also important from the clinicians’ perspectives of this region. As the review reports positive correlation between GDM and adverse pregnancy outcomes, it is important that clinicians also consider the possibilities of any adverse pregnancy outcomes while recommending the treatment regimens for GDM patients.

To the authors’ knowledge, as no other recent systematic reviews have been conducted summarizing the role of GDM on adverse pregnancy outcomes focusing in South Asia.^45^ This review is particularly important as it provides synthesized evidence from South Asian countries for policymakers and clinical as well as public health practitioners to enact effective initiatives to address the issue.

However, one should be cautious while interpreting the findings of the research as the quality of the included studies was relatively moderate^8^, ^9^, ^23^, ^25^, ^27^, ^28^ to poor.^24^, ^26^ Moreover, most of the included studies followed a prospective observational design and reported differences in the prevalence of adverse outcomes between GDM and non‐GDM group, but they did not report a causative association between GDM and adverse pregnancy outcomes. There was significant heterogeneity among selected studies in terms of participants’ age, BMI, dietary patterns, study design and the methods of diagnostic criteria and this could have affected the reliability and validity of the findings. It is to be noted that there were limited studies on the association between GDM and pregnancy outcomes in some of the South Asian countries including Afghanistan, Maldives and Bhutan.

## 5 CONCLUSION

Overall, the findings from the current systematic review suggests a positive association between GDM and adverse pregnancy outcomes in South Asian countries. The review provides valuable information for policymakers and practitioners to undertake effective initiatives to address the issue and to improve the GDM‐related care for reproductive aged women. The findings also indicate the need to undertake longitudinal studies to better understand the causative link between GDM and adverse pregnancy outcomes in South Asian countries.

## CONFLICT OF INTEREST

The authors report that there is no conflict of interest to disclose.

## AUTHOR CONTRIBUTIONS

SKM and KK conceptualized and designed the study. SKM, RDG, SA and KK conducted the formal analysis for the study. SKM, RDG, SA, KK and AAS contributed to writing the first draft of the manuscript. SP commented extensively on the draft of the manuscript to finalize. All authors read and approved the final version of the manuscript.

## References

[1] Kanguru L , Bezawada N , Hussein J , Bell J . The burden of diabetes mellitus during pregnancy in low‐and middle‐income countries: a systematic review. Glob Health Action. 2014;7(1):23987. 24990684 10.3402/gha.v7.23987 PMC4079934

[2] Pilliod RA , Page JM , Burwick RM , Kaimal AJ , Cheng YW , Caughey AB . The risk of fetal death in nonanomalous pregnancies affected by polyhydramnios. Am J Obstet Gynecol. 2015;213(3):410.e1‐410.e6. 25981851 10.1016/j.ajog.2015.05.022

[3] Berkowitz GS , Lapinski RH , Wein R , Lee D . Race/ethnicity and other risk factors for gestational diabetes. Am J Epidemiol. 1992;135(9):965‐973. 1595695 10.1093/oxfordjournals.aje.a116408

[4] Di Cianni G , Volpe L , Lencioni C , et al. Prevalence and risk factors for gestational diabetes assessed by universal screening. Diabetes Res Clin Pract. 2003;62(2):131‐137. 14581150 10.1016/j.diabres.2003.07.004

[5] Teh WT , Teede HJ , Paul E , Harrison CL , Wallace EM , Allan C . Risk factors for gestational diabetes mellitus: implications for the application of screening guidelines. Aust N Z J Obstet Gynaecol. 2011;51(1):26‐30. 21299505 10.1111/j.1479-828X.2011.01292.x

[6] Goedegebure EAR , Koning SH , Hoogenberg K , et al. Pregnancy outcomes in women with gestational diabetes mellitus diagnosed according to the WHO‐2013 and WHO‐1999 diagnostic criteria: a multicentre retrospective cohort study. BMC Pregnancy Childbirth. 2018;18(1):152. 29747601 10.1186/s12884-018-1810-5 PMC5946499

[7] Jawad F , Ejaz K . Gestational diabetes mellitus in South Asia: epidemiology. J Pak Med Assoc. 2016;66(9 Suppl 1):S5‐S7. 27582153

[8] Misra S , Das NK . Neonatal outcome in early and late gestational diabetes mellitus. J Nepal Paediatr Soc. 2017;37(1):41‐44.

[9] Saxena P , Tyagi S , Prakash A , Nigam A , Trivedi SS . Pregnancy outcome of women with gestational diabetes in a tertiary level hospital of North India. Indian J Community Med. 2011;36(2):120‐123. 21976796 10.4103/0970-0218.84130 PMC3180936

[10] Wells JCK , Pomeroy E , Walimbe SR , Popkin BM , Yajnik CS . The elevated susceptibility to diabetes in India: an evolutionary perspective. Front Public Health. 2016;4:145. 27458578 10.3389/fpubh.2016.00145 PMC4935697

[11] Schmidt CB , Voorhorst I , van de Gaar VHW ,, et al. Diabetes distress is associated with adverse pregnancy outcomes in women with gestational diabetes: a prospective cohort study. BMC Pregnancy Childbirth. 2019;19(1):223. 31269913 10.1186/s12884-019-2376-6 PMC6610799

[12] Dalfrà MG , Nicolucci A , Bisson T , Bonsembiante B , Lapolla A . Quality of life in pregnancy and post‐partum: a study in diabetic patients. Qual Life Res. 2012;21(2):291‐298. 21633879 10.1007/s11136-011-9940-5

[13] Silverman ME , Reichenberg A , Savitz DA , et al. The risk factors for postpartum depression: A population‐based study. Depress Anxiety. 2017;34(2):178‐187. 28098957 10.1002/da.22597 PMC5462547

[14] Perna R , Loughan AR , Le J , Tyson K . Gestational diabetes: long‐term central nervous system developmental and cognitive sequelae. Appl Neuropsychol Child. 2015;4(3):217‐220. 25265045 10.1080/21622965.2013.874951

[15] Rahman MA , Khan N , Rahman MM . Maternal anaemia and risk of adverse obstetric and neonatal outcomes in South Asian countries: a systematic review and meta‐analysis. Public Health Pract. 2020;1:100021. 10.1016/j.puhip.2020.100021 PMC9461600 36101702

[16] Ali SA , Tikmani SS , Saleem S , et al. Hemoglobin concentrations and adverse birth outcomes in South Asian pregnant women: findings from a prospective maternal and neonatal health registry. Reprod Health. 2020;17(2):1‐13. 33256770 10.1186/s12978-020-01006-6 PMC7706196

[17] Poudel S , Ghimire PR , Upadhaya N , Rawal L . Factors associated with stillbirth in selected countries of South Asia: a systematic review of observational studies. PLoS One. 2020;15(9):e0238938. 32936823 10.1371/journal.pone.0238938 PMC7494090

[18] Saleem T , Sajjad N , Fatima S , Habib N , Ali SR , Qadir M . Intrauterine growth retardation‐small events, big consequences. Ital J Pediatr. 2011;37(1):1‐4. 21899747 10.1186/1824-7288-37-41 PMC3177763

[19] Verma V , Vishwakarma RK , Nath DC , Khan HTA , Prakash R , Abid O . Prevalence and determinants of caesarean section in South and South‐East Asian women. PLoS One. 2020;15(3):e0229906. 32163440 10.1371/journal.pone.0229906 PMC7067459

[20] Liberati A , Altman DG , Tetzlaff J , et al. The PRISMA statement for reporting systematic reviews and meta‐analyses of studies that evaluate healthcare interventions: explanation and elaboration. BMJ. 2009;339:b2700. 19622552 10.1136/bmj.b2700 PMC2714672

[21] Effective Public Healthcare Panacea Project. Published Quality Assessment Tool for Quantitative Studies. https://www.ephpp.ca/quality‐assessment‐tool‐for‐quantitative‐studies/. Accessed May 18, 2020.

[22] Green S . Systematic reviews and meta‐analysis. Singapore Med J. 2005;46(6):270. 15902354

[23] Akhter J , Qureshi R , Rahim F , et al. Diabetes in pregnancy in Pakistani women: prevalence and complications in an indigenous south Asian community. Diabet Med. 1996;13(2):189‐191. 8641127 10.1002/(SICI)1096-9136(199602)13:2<189::AID-DIA32>3.0.CO;2-4

[24] Islam MN , Tazmin T , Siddika M , Bhuiyan MKJ . Morbidities and mortalities among infant of diabetic mother in a newly established SCANU of a tertiary care hospital, Bangladesh. J Nepal Paediatr Soc. 2015;35(3):253‐256.

[25] Jayawardane A , Patabendige M , Samaranayake D , et al. Hyperglycemia in pregnancy among South Asian women: A single tertiary care center experience from Colombo, Sri Lanka. Diabetes Res Clin Pract. 2018;145:138‐145. 29526683 10.1016/j.diabres.2018.02.029

[26] Goswami Mahanta T , Deuri A , Mahanta BN , et al. Maternal and foetal outcome of gestational diabetes mellitus in a rural block of Assam, India. Clin Epidemiol Glob Health. 2014;2(1):9‐15.

[27] Shefali AK , Kavitha M , Deepa R , Mohan V . Pregnancy outcomes in pre‐gestational and gestational diabetic women in comparison to non‐diabetic women–A prospective study in Asian Indian mothers (CURES‐35). J Assoc Physicians India. 2006;54:613‐618. 16941791

[28] Wahi P , Dogra V , Jandial K , et al. Prevalence of gestational diabetes mellitus (GDM) and its outcomes in Jammu region. J Assoc Physicians India. 2011;59(4):227‐230. 21755759

[29] Usta A , Usta CS , Yildiz A , et al. Frequency of fetal macrosomia and the associated risk factors in pregnancies without gestational diabetes mellitus. Pan Afr Med J. 2017;26:62. 28451039 10.11604/pamj.2017.26.62.11440 PMC5398855

[30] Vambergue A , Fajardy I . Consequences of gestational and pregestational diabetes on placental function and birth weight. World J Diabetes. 2011;2(11):196. 22087356 10.4239/wjd.v2.i11.196 PMC3215769

[31] Kamana KC , Shakya S , Zhang H . Gestational diabetes mellitus and macrosomia: a literature review. Ann Nutr Metab. 2015;66(Suppl. 2):14‐20. 10.1159/000371628 26045324

[32] Lazer S , Biale Y , Mazor M , Lewenthal H , Insler V . Complications associated with the macrosomic fetus. J Reprod Med. 1986;31(6):501. 3735262

[33] Seghieri G , Anichini R , De Bellis A , Alviggi L , Franconi F , Breschi MC . Relationship between gestational diabetes mellitus and low maternal birth weight. Diabetes Care. 2002;25(10):1761‐1765. 12351474 10.2337/diacare.25.10.1761

[34] Odar E , Wandabwa J , Kiondo P . Maternal and fetal outcome of gestational diabetes mellitus in Mulago Hospital, Uganda. Afr Health Sci. 2004;4(1):9‐14. 15126187 PMC2141655

[35] Wang M , Athayde N , Padmanabhan S , Cheung NW . Causes of stillbirths in diabetic and gestational diabetes pregnancies at a NSW tertiary referral hospital. Aust N Z J Obstet Gynaecol. 2019;59(4):561‐566. 30663043 10.1111/ajo.12936

[36] Dudley DJ . Diabetic‐associated stillbirth: incidence, pathophysiology, and prevention. Clin Perinatol. 2007;34(4):611‐626. 18063109 10.1016/j.clp.2007.09.003

[37] Voormolen DN , de Wit L , van Rijn BB , et al. Neonatal hypoglycemia following diet‐controlled and insulin‐treated gestational diabetes mellitus. Diabetes Care. 2018;41(7):1385‐1390. 29654142 10.2337/dc18-0048

[38] Bryson CL , Ioannou GN , Rulyak SJ , Critchlow C . Association between gestational diabetes and pregnancy‐induced hypertension. Am J Epidemiol. 2003;158(12):1148‐1153. 14652299 10.1093/aje/kwg273

[39] Li L‐J , Aris IM , Su LL , et al. Effect of gestational diabetes and hypertensive disorders of pregnancy on postpartum cardiometabolic risk. Endocr Connect. 2018;7(3):433‐442. 29444890 10.1530/EC-17-0359 PMC5834770

[40] Boriboonhirunsarn D , Waiyanikorn R . Emergency cesarean section rate between women with gestational diabetes and normal pregnant women. Taiwan J Obstet Gynecol. 2016;55(1):64‐67. 26927251 10.1016/j.tjog.2015.08.024

[41] Montoro MN , Kjos SL , Chandler M , Peters RK , Xiang AH , Buchanan TA . Insulin resistance and preeclampsia in gestational diabetes mellitus. Diabetes Care. 2005;28(8):1995‐2000. 16043744 10.2337/diacare.28.8.1995

[42] Innes K , Wimsatt J . Pregnancy‐induced hypertension and insulin resistance, evidence for a connection. Acta Obstet Gynecol Scand. 1999;78(4):263‐284. 10203292

[43] Ness RB , Roberts JM . Heterogeneous causes constituting the single syndrome of preeclampsia: a hypothesis and its implications. Am J Obstet Gynecol. 1996;175(5):1365‐1370. 8942516 10.1016/s0002-9378(96)70056-x

[44] Mak JKL , Lee AH , Pham NM , et al. Gestational diabetes incidence and delivery outcomes in Western China: a prospective cohort study. Birth. 2019;46(1):166‐172. 30216525 10.1111/birt.12397

[45] Nguyen CL , Pham NM , Binns CW , Duong DV , Lee AH . Prevalence of gestational diabetes mellitus in eastern and southeastern Asia: a systematic review and meta‐analysis. J Diabetes Res. 2018;2018:6536974. 29675432 10.1155/2018/6536974 PMC5838488

